# Automated diabetic retinopathy detection in smartphone-based fundus photography using artificial intelligence

**DOI:** 10.1038/s41433-018-0064-9

**Published:** 2018-03-09

**Authors:** Ramachandran Rajalakshmi, Radhakrishnan Subashini, Ranjit Mohan Anjana, Viswanathan Mohan

**Affiliations:** grid.410867.c 0000 0004 1805 2183 Dr. Mohan’s Diabetes Specialities Centre & Madras Diabetes Research Foundation, WHO Collaborating Centre for Noncommunicable Diseases Prevention and Control, IDF Centre of Excellence in Diabetes Care & ICMR Centre for Advanced Research on Diabetes, Chennai, Tamil Nadu India

**Keywords:** Outcomes research, Retinal diseases

## Abstract

**Objectives:**

To assess the role of artificial intelligence (AI)-based automated software for detection of diabetic retinopathy (DR) and sight-threatening DR (STDR) by fundus photography taken using a smartphone-based device and validate it against ophthalmologist’s grading.

**Methods:**

Three hundred and one patients with type 2 diabetes underwent retinal photography with Remidio ‘Fundus on phone’ (FOP), a smartphone-based device, at a tertiary care diabetes centre in India. Grading of DR was performed by the ophthalmologists using International Clinical DR (ICDR) classification scale. STDR was defined by the presence of severe non-proliferative DR, proliferative DR or diabetic macular oedema (DME). The retinal photographs were graded using a validated AI DR screening software (EyeArt^TM^) designed to identify DR, referable DR (moderate non-proliferative DR or worse and/or DME) or STDR. The sensitivity and specificity of automated grading were assessed and validated against the ophthalmologists’ grading.

**Results:**

Retinal images of 296 patients were graded. DR was detected by the ophthalmologists in 191 (64.5%) and by the AI software in 203 (68.6%) patients while STDR was detected in 112 (37.8%) and 146 (49.3%) patients, respectively. The AI software showed 95.8% (95% CI 92.9–98.7) sensitivity and 80.2% (95% CI 72.6–87.8) specificity for detecting any DR and 99.1% (95% CI 95.1–99.9) sensitivity and 80.4% (95% CI 73.9–85.9) specificity in detecting STDR with a kappa agreement of *k* = 0.78 (*p* < 0.001) and *k* = 0.75 (*p* < 0.001), respectively.

**Conclusions:**

Automated AI analysis of FOP smartphone retinal imaging has very high sensitivity for detecting DR and STDR and thus can be an initial tool for mass retinal screening in people with diabetes.

## Introduction

All individuals with diabetes, irrespective of the type of diabetes, require regular and repetitive annual retinal screening for early detection and timely treatment of diabetic retinopathy (DR), particularly sight-threatening diabetic retinopathy (STDR) [1, 2]. Screening for retinopathy is conventionally done through fundus examination by ophthalmologists or retinal colour photography using conventional mydriatic or non-mydriatic fundus cameras by optometrists or trained eye technicians [3]. Smartphone-based retinal imaging has emerged as one of the recent cost-effective ways of screening for retinopathy in the community [3–5]. However, till date irrespective of the type of fundus camera used, the retinal images had to be graded for the presence and severity of DR by ophthalmologists (retinal specialists) or trained graders [3–5].

Given the alarming increase in the number of people with diabetes and shortage of trained retinal specialists and graders of retinal photographs, an automated approach involving a computer-based analysis of the fundus images would reduce the burden of the health systems in screening for DR [6–8]. There is hence an increasing interest in the recent past in the development of automated analysis software using computer machine learning/artificial intelligence (AI)/deep neuronal learning for analysis of retinal images in people with diabetes [7, 9]. AI is simulation of human intelligence by a software/machine. It is a specialised field which is based on teaching the machine to recognise specific patterns. It has been used for different kinds of technical tasks including accurate classification of high-resolution images. AI for detection and classification of DR happens by providing thousands of retinal images of varying grades of DR to the system for learning. The machine after being exposed to a lot of annotated retinal images learns to grade DR by itself. Some studies done in the recent past have shown that AI could be used to grade retinal images taken using the conventional fundus cameras and determine which patients with DR need referral to the ophthalmologist [7–9]. Many of the AI algorithms have been trained and developed using retinal images from high-quality conventional fundus cameras.

To our knowledge, assessment of the use of AI along with smartphone-based fundus photography has not been done so far. The aim of this study was to evaluate the usefulness of an automated AI-based interpretation of smartphone-based fundus photography system for screening at a physician clinic (without the algorithm being trained specifically on retinal images from smartphone fundus photography). This paper studies the accuracy of the automated DR software in DR detection and screening for STDR using a previously validated smartphone-based retinal imaging system.

## Methods

Three hundred and one patients with type 2 diabetes, aged 18 years and above, undergoing treatment for diabetes at a tertiary care diabetes hospital in Chennai (formerly Madras) in southern India, with varying duration of diabetes underwent retinal colour photography (fundus photography) at the eye department as a part of regular retinal screening for DR. A pilot study was conducted with retinal images of 50 patients to assess the sensitivity and specificity of automated DR detection using the EyeArt^TM^ software. The sample size for this study was calculated based on the results of the pilot phase. A written informed consent was obtained from all participants and the study was approved by the Ethical Committee of the Madras Diabetes Research Foundation.

**Fig. 1:**
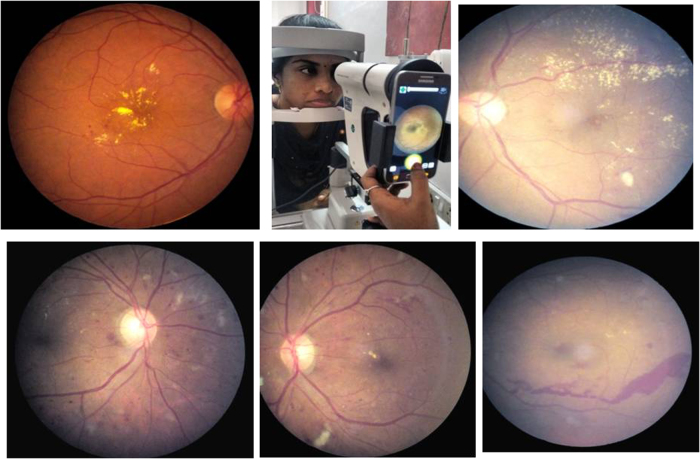
Remidio Fundus on Phone—retinal imaging

After preliminary eye examination, the pupils were dilated with tropicamide eye drops after ruling out any history of allergy to the dilating eye drops. The retinal photographs were acquired using Remidio Fundus on Phone (FOP), a smartphone-based imaging device (Remidio Innovative Solutions Pvt. Ltd, Bangalore, India). It is a portable fundus camera that consists of an annular illumination design that eliminates corneal reflection, which mates with any commercially available smartphone to acquire retinal photographs [4]. The FOP provides a 45° field of view for fundus imaging and can be used in a handheld mode or be fit onto any standard slitlamp as shown in Fig. 1. Four fields were captured in each eye on the FOP camera: macula centred, disc centred, superior-temporal and inferior-temporal quadrants of the retina. Retinal photographs were coded using an identification number and assessed in a masked manner for the presence and severity of DR in order to minimise any possible bias. The photographs were graded by two ophthalmologists (retina specialists) who were masked to the patient’s identity and clinical diagnosis. The kappa agreement between the grading of the two ophthalmologists was *k* = 0.89. Any disagreement in the retinopathy grading between the two graders was adjudicated by a third retina specialist whose DR grading was taken as final. The grading of retinopathy was done based on the International Clinical Diabetic Retinopathy (ICDR) severity scale [10]. The ICDR severity scales provides a classification of five stages of DR as follows: (1) no apparent retinopathy—no abnormalities; (2) mild non-proliferative DR (NPDR)—microaneurysms only; (3) moderate NPDR—more than just microaneurysms, but less than severe NPDR; (4) severe NPDR—one or more of the following: (i) more than 20 intra-retinal haemorrhages in each of four quadrants, (ii) definite venous beading in two or more quadrants, (iii) prominent intra-retinal microvascular abnormality in one or more quadrants; (5) proliferative DR (PDR)—retinal neovascularisation with or without vitreous/preretinal haemorrhage [10]. Sight-threatening DR (STDR) was defined by the presence of severe NPDR, PDR and/or diabetic macular oedema (DME)/clinically significant macular oedema (CSME) [8]. Photographs were graded and assigned a retinopathy level and the final diagnosis for each patient was determined from the level of DR of the worse eye using ICDR severity scale. Figure 1 shows some Remidio FOP retinal images of varying severity of DR.

The retinal images after masking the identity of the patient and the diagnosis were shared with EyeNuk Inc. (Los Angeles, CA, USA) for the automated analysis with the EyeArt^TM^ software (EyeNuk Inc., Los Angeles, CA). EyeArt^TM^ is a computerised, cloud-based software that can automatically analyse retinal images and provide DR severity and screening recommendation by automatically detecting the presence, size, position and number of retinal lesions. In addition to DR severity level on the ICDR severity scale, presence/absence of surrogate markers for CSME which is defined by the presence of hard exudates within one disc diameter of the centre of the macula are also reported by the algorithm. EyeArt’s core analysis engine contains DR analysis algorithms including those for image enhancement, interest region identification, descriptor computation, in conjunction with an ensemble of deep artificial neural networks for DR classification, and detection of CSME surrogate markers [9]. The EyeArt AI algorithm has been clinically AI trained and validated using retinal images of 78,685 patients taken using conventional desktop mydriatic fundus cameras-obtained from the EyePACS database [11]. Referable diabetic eye disease (RDR) by EyeArt is defined as the presence of (i) moderate NPDR or higher and/or (ii) CSME [7, 9]. A “no refer” recommendation was provided for patients with no apparent signs of DR or signs of mild DR without CSME [11].

A total of 2408 Remidio FOP retinal images of 301 patients were shared through the cloud with EyeNuk for this study. EyeArt^TM^ software (version v2.1.0) which was used in this study provided a patient wise diagnosis of DR.

### Statistical analysis

All statistical analyses were performed using SAS statistical package version 9.2. The sensitivity and specificity of EyeArt algorithm for detecting DR and diagnosing referable/sight-threatening DR were calculated by generating 2 × 2 tables taking the ophthalmologist grading as the reference standard. Positive predictive value (PPV) was defined as the probability of the presence of disease given a positive test result and negative predictive value (NPV) was defined as the probability of the absence of disease given a negative test result. 95% confidence intervals (CIs) were used for sensitivity, specificity, PPV and NPV. The degree of agreement between automated analysis and manual (ophthalmologist) grading was quantified and assessed using the kappa (*ĸ*) statistics. For all statistical tests, *p-*value <0.05 was considered significant.

## Results

**Fig. 2:**
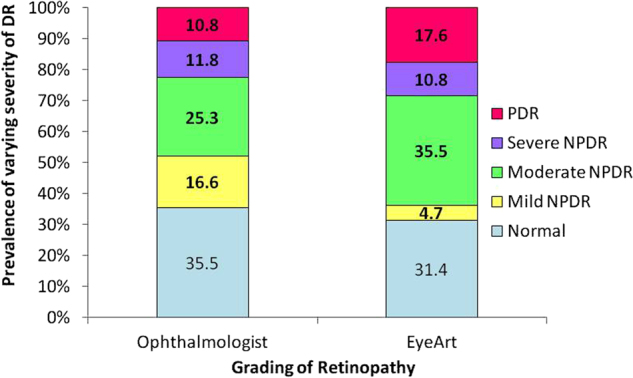
Comparison of diabetic retinopathy (DR) severity between ophthalmologist and EyeArt software grading (*n* = 296)

DR grading was done by the ophthalmologists for Remidio FOP retinal images of 301 (602 eyes) type 2 diabetes patients and a total of 2408 FOP retinal images were shared with EyeNuk Inc. The EyeArt^TM^ AI algorithm provided diagnosis for retinal images of 296 patients. Retinal photographs of five patients were deemed to be of inadequate quality for automated analysis when the media was unclear due to cataract or asteroid hyalosis or vitreous haemorrhage. Thus the statistical analysis and the comparisons were done for 296 patients. DR was detected by the ophthalmologist in 191 (64.5%) patients by grading the smartphone-based retinal images while the EyeArt software detected DR in 203 (68.3%) of the 296 patients. The varying grades of DR in the two modes of retinopathy grading based on ICDR are depicted in Fig. 2. Based on manual grading by the ophthalmologist 142 (48%) patients had RDR (moderate NPDR and above), while based on the EyeArt grading, 189 (63.9%) patients had RDR. STDR was detected in 112 (37.8%) by the ophthalmologist and in 146 (49.3%) patients by EyeArt algorithm.

**Fig. 3:**
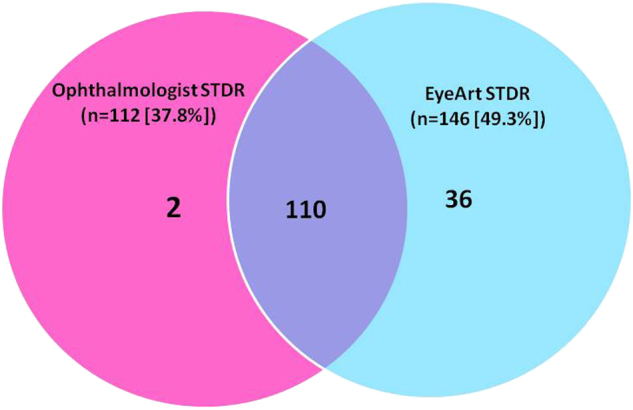
Venn diagram showing the overlap comparison of sight-threatening diabetic retinopathy (STDR) between manual (Ophthalmologist) and Software (EyeArt^TM^) grading (*n* = 296)

Figure 3 shows the Venn diagram of the STDR identified by the ophthalmologist vs. the software and the overlap and matched diagnosis of STDR observed in 110 patients.

The sensitivity and specificity for detecting any DR, DME, PDR, STDR, RDR, by EyeArt software against the ophthalmologist grading as the reference standard and the degree of agreement between two is shown in Table 1. The automated software correctly identified 95.8% of patients with retinopathy and 80.2% of patients without retinopathy. EyeArt showed 95.8% (95% CI 92.9–98.7) sensitivity and 80.2% (95% CI 72.6–87.8) specificity for detecting DR and 99.1% (95% CI 95.1–99.9) sensitivity and 80.4% (95% CI 73.9–85.9) specificity in diagnosing STDR. The degree of agreement between automated and ophthalmologist grading for any DR was 0.78 (*p* < 0.001) and for STDR was 0.75 (*p* < 0.001) using the kappa (*ĸ*) statistics. The sensitivity for referable DR (RDR) was 99.3% and the specificity was 68.8% and the ĸ agreement between the human vs. machine grading for RDR was 0.67 (*p* < 0.001). As the sensitivity values were high, the PPV and the NPV were also calculated as shown in Table 1. The PPV for detection of DR, STDR and RDR was 89.7% (95% CI 85.5–93.8), 75.3% (95% CI 68.4–82.3) and 74.6% (95% CI 68.4–80.8), respectively.

**Table 1:** Sensitivity, specificity, positive predictive value (PPV), negative predictive value (NPV) and kappa agreement of EyeArt for detection of any varying degrees of DR with ophthalmologist grading as reference standard

Retinopathy	Sensitivity, % (95% CI)	Specificity, % (95% CI)	PPV (95% CI)	NPV (95% CI)	Kappa (95% CI)	*p-*value
Any DR	95.8 (92.9, 98.7)	80.2 (72.6, 87.8)	89.7 (85.5, 93.8)	91.4 (85.7, 97.1)	0.78 (0.71, 0.86)	<0.001
DME	97 (91.5, 99.4)	75.8 (69.7, 81.8)	67.4 (59.7, 75.0)	98.0 (94.3, 99.6)	0.66 (0.58, 0.74)	<0.001
PDR	78.1 (63.8, 83.3)	89.8 (86.1, 93.4)	48.1 (34.5, 61.7)	97.1 (95.0, 99.2)	0.53 (0.4, 0.67)	<0.001
STDR	99.1 (95.1, 99.9)	80.4 (73.9, 85.9)	75.3 (68.4, 82.3)	99.3 (96.3, 100)	0.75 (0.67, 0.83)	<0.001
RDR	99.3 (96.1, 99.9)	68.8 (61.5, 76.2)	74.6 (68.4, 80.8)	99.1 (97.2, 100)	0.67 (0.59, 0.75)	<0.001

*Any DR* diabetic retinopathy (any stage), *DME* diabetic macular oedema, *PDR* proliferative diabetic retinopathy, *STDR* sight-threatening diabetic retinopathy (severe NPDR, PDR and DME), *RDR* referable diabetic retinopathy (moderate NPDR and above and/or presence of clinically significant macular oedema [CSME])

## Discussion

This study has assessed the role of automated AI algorithm in detection of DR using a low-cost smartphone-based imaging device. To our knowledge, this is the first time that an automated AI DR screening software has been tested for its accuracy for smartphone-based retinal imaging.

Regular retinal screening for all people with diabetes is still an unmet need in most countries especially in poor developing countries. Retinal photography with grading and interpretation by ophthalmologists/retina specialists and trained graders is widely accepted for screening for DR [4, 12, 13]. However, availability of retina specialists/trained retinal graders is a major limitation in most countries, including India. Even when available, there could be a time delay in graders submitting their DR grading and advice due to their busy schedule. This leads, not only to delayed interpretation, but also loss to follow-up, miscommunication, and delay in proceeding for management of STDR [14]. Deep learning/AI for detection of DR happens by machine learning by providing thousands of retinal images of varying grades of DR to the system for learning [7, 8]. The need and efforts for a comprehensive automated method of DR screening have made good progress using image classification, pattern recognition and machine learning techniques [15, 16].

We report here, a high sensitivity for detection of DR, STDR and RDR (above 95% for DR, 99% for STDR and RDR) using the EyeArt software when used on retinal images taken with FOP. This is similar to the high sensitivity in the Google AI algorithm which showed a high sensitivity and specificity for RDR when used on conventional retinal photography (sensitivity of 97.5% and specificity of 93.4% in the EYEPACS-1 and 96.1% sensitivity and 93.9% specificity for Messidor-2 set) [7]. One of the main reasons for the lower specificity for RDR in our study was because of higher estimation of Moderate NPDR with the images assessed by the algorithm. Some non-DR retinal lesions like drusen, RPE atrophic patch, a retinal telangiectatic vessel at macula, RPE hypertrophy, tessellated fundus and retinal vein occlusion were the causes of false positives. However, these non-DR retinal lesions also need advice from the ophthalmologist/retina specialist and hence they were not truly false positives. The specific impact of formal training on retinal images of varying DR severity from Remidio FOP/smartphone-based fundus cameras on the algorithm could probably lead to higher specificity for RDR detection in the future.

A recent study done by EyeNuk with retinal images taken with traditional desktop fundus cameras showed that EyeArt’s sensitivity for DR screening sensitivity was 91.7% (95% CI: 91.3–92.1%) and specificity was 91.5% (95% CI: 91.2–91.7%) [17]. They also showed that the sensitivity for detecting treatable diabetic eye disease (STDR) was 98.5%, i.e. of the 470 eyes with treatable DED as per reference standard, 465 eyes were correctly provided “refer” recommendations by the algorithm [11]. This is similar to the sensitivity for detecting DR and STDR in our study using smartphone-based retinal images.

In a very recent major study publication on validation of deep learning (AI) by Ting et al. [18] done in Singapore with multiple retinal images taken with conventional fundus cameras from multi-ethnic cohorts of people with diabetes, their algorithm showed a high sensitivity and specificity for identifying DR and other eye diseases like age-related macular degeneration. The sensitivity and specificity for RDR was 90.5% (95% CI 87.3–93.0%) and 91.6% (95% CI 91.0–92.2%) respectively and for STDR the sensitivity was 100% (95% CI 94.1–100.0%) and the specificity was 91.1% (95% CI 90.7–91.4%) in their study [18]. In our study done using FOP smartphone-based retinal images, we have reported a similar high sensitivity for RDR and STDR detection.

The IRIS (intelligent retinal imaging system), an automated teleretinal DR screening programme, compared non-mydriatic retinal images with a standard data set images from Early Treatment Diabetic Retinopathy Study (ETDRS) and gave suggestions for referral. Any patient with severe NPDR or more advanced disease was considered suitable for the referral [8]. IRIS screening programme had a good sensitivity and a low false-negative rate [8]. The sensitivity of the IRIS algorithm in detecting STDR compared with the reading centre interpretation was 66.4% (95% CI 62.8–69.9) with a false-negative rate of 2% and the specificity was 72.8% (95% CI 72.0–73.5). In our study the sensitivity for STDR was 99.1% and the specificity was 80.4%. The lower sensitivity with IRIS was possibly because the system used non-mydriatic retinal images.

It is of interest that, in this study, despite no formal machine learning/training of the algorithm on the images from Remidio FOP, the EyeArt solution was able to grade images for STDR with a high sensitivity of 99.1% and specificity of 80.4%. The results reported in this paper are in agreement with our earlier study that reported a high level of agreement when comparing the ophthalmologist grading of retinal images from the FOP and the Zeiss FF450 conventional mydriatic fundus camera for retinopathy screening [4]. The EyeArt software is already trained on retinal images from conventional fundus cameras like the Zeiss FF450 [9]. It is thus reassuring that the AI algorithm also works well on the FOP smartphone-based imaging system.

Use of AI to analyse retinal images is appealing as it fits in with the current trend of tele-ophthalmology and telemedicine [13, 19]. Automated DR grading softwares have potential benefits of efficiency, reproducibility and early detection of DR happening at the physician level and thus would be useful in reducing the burden to the health systems in screening of the increasing number of people with diabetes [16, 20]. Only those who have sight-threatening DR and referable DR would need to meet the ophthalmologist/ retina specialist. Urgent referral of patients with sight-threatening DR to the retina specialist for further evaluation and treatment is essential, especially since DR affects people with diabetes during their prime productive years of life [21].

### Strengths of the study

To the best of our knowledge, this is the first study that has looked at the role and accuracy of automated AI-based DR analysis in smartphone-based retinal imaging. In this study, as the EyeArt^TM^ software had already been validated [11] and the Remidio FOP imaging system also had been validated for DR screening [4], quality automated DR detection in this study was possible without formal training with large number of FOP retinal images for deep learning. Automated softwares using the AI like the EyeArt along with telemedicine can enable faster real-time screening of large number of people with diabetes. Smartphone retinal colour photography combined with an automated detection system can ideally result in models with potential for cost-effective routine clinical use by the primary care physicians. Admittedly, further work is needed before recommending its regular use in eye care.

### Limitations of the study

The sample size is relatively small when compared to other studies that have recently assessed role of AI in DR. However, as this study has not used any images from Remidio FOP to train EyeArt, large datasets were not necessary. Although the AI algorithm could replicate manual grading by ophthalmologists, it could not overcome physical limitations, such as inability to acquire photographs in some patients due to poor mydriasis, poor image quality due to media opacities like cataract [22]. The conclusions from this study cannot necessarily be extended to all smartphone-based imaging devices, unless they have been independently validated for performance in DR screening.

### Conclusions of the study

In summary, an AI-based grading algorithm in combination with validated smartphone-based imaging of diabetic patients can be used to reliably and accurately screen patients for sight-threatening DR who could then be referred to the retina specialist for further evaluation and treatment. As patients will anyway be referred to the ophthalmologist, false-positive cases can be excluded by them and those who need treatment can be given the appropriate therapy. The automated analysis algorithm installed inside a low-cost sleek fundus camera can also be very useful for mass scale DR screening programmes particularly in remote areas of poorly developed countries where trained personnel may not be available.

### Summary

#### What was known before

- Retinal images had to be graded for the presence and severity of DR by ophthalmologists (retinal specialists) or trained graders.
- Studies done in the recent past have shown that Artificial intelligence (AI) could be used to grade retinal images taken using the conventional fundus cameras and determine which patients with DR need referral to the ophthalmologist.

#### What this study adds

- Assessment of the use of Artificial intelligence (AI) along with smartphone-based fundus photography for Diabetic retinopathy (DR) detection and classification has not been done so far.
- Automated AI analysis of smartphone retinal imaging has very high sensitivity for detecting DR and sight-threatening DR and thus can be an initial tool for mass retinal screening in people with diabetes.
